# Does oscillation size matter? Impact of added resistance on the cerebral pressure‐flow Relationship in females and males

**DOI:** 10.14814/phy2.15278

**Published:** 2022-05-17

**Authors:** Kailey T. Newel, Joel S. Burma, Joseph Carere, Courtney M. Kennedy, Jonathan D. Smirl

**Affiliations:** ^1^ Cerebrovascular Concussion Lab Faculty of Kinesiology University of Calgary Alberta Canada; ^2^ Sport Injury Prevention Research Centre Faculty of Kinesiology University of Calgary Calgary Alberta Canada; ^3^ Hotchkiss Brain Institute University of Calgary Calgary Alberta Canada; ^4^ Integrated Concussion Research Program University of Calgary Calgary Alberta Canada; ^5^ Faculty of Health and Exercise Science University of British Columbia Kelowna British Columbia Canada; ^6^ Alberta Children's Hospital Research Institute University of Calgary Calgary Alberta Canada; ^7^ Human Performance Laboratory Faculty of Kinesiology University of Calgary Calgary Alberta Canada; ^8^ Libin Cardiovascular Institute of Alberta University of Calgary Alberta Canada

**Keywords:** cerebral blood flow regulation, doppler ultrasound, dynamic cerebral autoregulation, resistance exercise, squat‐stand maneuvers

## Abstract

Sinusoidal squat‐stand maneuvers (SSM) without resistance have been shown to produce ~30–50 mmHg swings in mean arterial pressure which are largely buffered in the brain via dynamic cerebral autoregulation (dCA). This study aimed to further elucidate how this regulatory mechanism is affected during SSM with added resistance (~20% bodyweight). Twenty‐five participants (sex/gender: 13 females/12 males) completed two bouts of 5‐min SSM for both bodyweight and resistance conditions (10% bodyweight in each arm) at frequencies of 0.05 Hz (20‐s squat/stand cycles) and 0.10 Hz (10‐s squat/stand cycles). Middle and posterior cerebral artery (MCA/PCA) cerebral blood velocities were indexed with transcranial Doppler ultrasound. Beat‐to‐beat blood pressure (BP) was quantified via finger photoplesmography. Transfer function analysis was employed to quantify dCA in both cerebral arteries across the cardiac cycle (diastole, mean, and systole). Two‐by‐two Analysis of Variance with generalized eta squared effect sizes were utilized to determine differences between resistance vs. bodyweight squats and between sexes/genders. Absolute mean and diastolic BP were elevated during the resistance squats (*p* < 0.001); however, only the BP point‐estimate power spectrum densities were augmented at 0.10 Hz (*p* < 0.048). No differences were noted for phase and gain metrics between bodyweight and resistance SSM (*p* > 0.067); however, females displayed attenuated systolic regulation (*p* < 0.003). Despite augmented systemic BP during resistance SSM, the brain was effective at buffering the additional stress to mitigate overperfusion/pressure. Females displayed less dCA regulation within the systolic aspect of the cardiac cycle, which may be associated with physiological underpinnings related to various clinical conditions/presentations.

## Graphical abstract

Dynamic cerebral autoregulation (dCA) has been demonstrated to effectively buffer ~30–50 mmHg swings in blood pressure, albeit it is unknown if this regulatory mechanism remains intact during larger blood pressure oscillations. During squat‐stand maneuvers with an added resistance (20% total body mass), the brain remained effective at dampening blood pressure to avoid overperfusion. Females displayed less buffering compared to their male counterparts, specifically within the systolic aspect of the cardiac cycle during 0.10 Hz squat‐stand maneuvers.

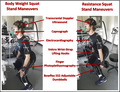

## 1 INTRODUCTION

The human brain has a constant and high metabolic demand (i.e., ~15%–20% resting oxygen consumption) but very limited substrate storage (Paulson et al., 1990; Williams & Leggett, 1989; Willie et al., 2014). In order to maintain optimal functionality, the brain requires adequate blood perfusion via the coordination of several key regulatory mechanisms (i.e., blood pressure [BP], carbon dioxide, neural demand, autonomic control, and cardiac output; Paulson et al., 1990; Willie et al., 2014). The former process describes the intrinsic ability of the cerebrovasculature to regulate blood perfusion/flow, with respect to alterations in BP (i.e., cerebral autoregulation [CA]; Brassard et al., 2021; Paulson et al., 1990; Zhang et al., 1998). There is increasing evidence demonstrating the cerebral pressure‐flow relationship has a very limited plateau region (~10 mmHg) where cerebral blood flow (CBF) is relatively stable and outside of this window, small changes in blood pressure will cause parallel fluctuations in CBF (Brassard et al., 2021; Claassen et al., 2021). Moreover, dynamic cerebral autoregulation (dCA) functions as a high‐pass filter, where oscillations in BP greater than 0.20 Hz occur too rapidly for the cerebrovascular to react and thus, these BP swings are passed through to the cerebral arterioles relatively unimpeded (Zhang et al., 1998). However, oscillations slower than <0.20 Hz are largely dampened and buffered by the cerebrovasculature (Zhang et al., 1998). Conclusively, an intact dCA is an imperative survival mechanism to mitigate against under‐ and over‐perfusion of the cerebrovasculature, which in turn protects against cerebrovascular accidents (e.g., ischemic stroke, hemorrhagic stroke, aneurysms, etc.; Aries et al., 2010; Lidington et al., 2021).

Previous support for the use of driven methods to quantify dCA has highlighted that challenging the cerebrovascular system by inducing transient oscillations in BP are the most robust approach for assessing cerebrovascular buffering capabilities (Simpson & Claassen, 2018). This is due to two reasons: (1) the clinical and physiological relevance for everyday tasks (e.g., bending over, walking upstairs, defecating, etc.) and (2) greater certainty there is a relationship between BP and CBF (Simpson & Claassen, 2018). While there is no universally, agreed‐upon “gold‐standard” approach to quantify dCA, squat‐stand maneuvers (SSM) are currently the most robust and reliable method to cause large and consistent blood pressure oscillations (~30–50 mmHg; Burma et al., 2020a; Claassen et al., 1985; Smirl et al., 1985). Further, previous work with SSM has noted the importance of delineating not just the cerebral pressure‐flow relationship to mean BP as an input, but also examining the diastolic and systolic aspects as well (Burma et al., 2020a; Burma, Copeland, Macaulay, Khatra, Wright, et al., 2020; Smirl et al., 2018; Wright et al., 2018). This is due to the differential buffering capacities between diastole and systole, where the former responds in a more pressure‐passive nature and thus, has a lesser buffering capacity (Burma et al., 2020a; Burma, Copeland, Macaulay, Khatra, Wright, et al., 2020; Smirl et al., 2018). Conversely, greater buffering is seen within the systolic aspect of the cardiac cycle, which is likely a protective mechanism to mitigate against increased systemic BP stressors (Smirl et al., 2018).

Of interest, maximal resistance leg press and squat repetitions are capable to produce mean arterial pressure (MAP) values exceeding 300–350 mmHg (MacDougall et al., 1985; Palatini et al., 1989). However, it is unknown the extent cerebral autoregulation remains intact during the ephemeral increases in MAP associated with maximal resistance exercise. The paucity of studies examining dCA *during* maximal resistance exercise is likely attributable to the fact a recording duration of 5 min is recommended in order to obtain valid and reliable dCA transfer function analysis (TFA) estimates (Burma et al., 2021). As weight‐bearing exercise primarily relies upon the phosphocreatine system to produce adenosine triphosphate (Hargreaves & Spriet, 2020), resistance exercise can only be sustained for few repetitions. This poses challenges when quantifying the cerebral‐pressure flow relationship during resistance exercise. Nevertheless, understanding the precise regulatory mechanisms of the brain is clinically relevant, as due to the Monroe‐Kellie Doctrine, the contents within the cranium have a constant volume, where an increase in CBF secondary to increased arterial and intracranial arterial pressure could lead to herniation of the brain (Wilson, 2016). Hence, engaging in heavy resistance exercise, where dCA impairments underly the pathophysiological changes of a given condition, may lead to detrimental and/or prolonged outcomes. Therefore, delineating the brain's mechanistic processes due to BP perturbations experienced on a daily basis (Simpson & Claassen, 2018), may help facilitate prevention strategies/training programs to inform recovery from brain injuries and potentially minimize the number of individuals experiencing cerebrovascular accidents. It is imperative to note, the previous dCA research has trended towards male‐dominated samples, which may not be universally generalizable. Furthermore, the pathogenesis and outcomes of various diseases/disorders have been demonstrated to be affected by chromosomal sex. Conclusively, understanding the regulatory differences in healthy populations regarding chromosomal sex may help understand the extent clinical treatment needs to vary depending upon the individual.

Therefore, the objective of this paper was two‐fold: (1) to elucidate the extent the brain is able to regulate changes in BP during SSM with added resistance across the cardiac cycle and (2) to delineate the similarities/differences between females and males within the cerebral pressure‐flow relationship during augmented cerebral stress. It was hypothesized there would be greater changes in systemic BP during the weighted SSM, which would translate to impaired TFA metrics (i.e., reduced phase and greater gain); and females would display attenuated dCA compared to their male counterparts (Favre & Serrador, 2019; Labrecque, Rahimaly, et al., 2019).

## 2 MATERIAL AND METHODS

### 2.1 Participants

A convenience sample of 25 participants were recruited from the university community. All subjects stated their chromosomal sex and self‐identified gender, which were the same, and therefore the sex/gender metrics will be presented as female/male throughout the manuscript. Out of the 25 participants, 13 were female (24.5 ± 3.6 years and body mass index [BMI]: 24.5 ± 3.6 kg/m^2^) and 12 were male participants (26.9 ± 4.6 years and BMI: 26.9 ± 4.6 kg/m^2^). No participants reported a history of any cerebrovascular, neurological, musculoskeletal, and/or respiratory diseases. Participants were instructed to refrain from exercise for a minimum of 6 h prior to data collection (Burma, Copeland, Macaulay, Khatra, Wright, et al., 2020) as well as abstain from alcohol, caffeine, smoking, and vaping for at least 8 h (Ainslie et al., 2007; Smirl et al., 1985); and food for 2 h prior to data collection (Baak, 2008; Burma et al., 2020b; Lefrandt et al., 2000).

### 2.2 Instrumentation

A transcranial Doppler ultrasound (Doppler Box, DWL USA Inc) was employed to index cerebral blood velocity (CBV) through the transtemporal window for the right middle cerebral artery (MCA) and left posterior cerebral artery (PCA; Willie et al., 2011). Once the MCA and PCA were identified and confirmed through visual and carotid compression checks, the probes were locked in place with a fitted headframe (DWL USA Inc). Heart rate based upon R‐R intervals was measured through a 3‐lead electrocardiogram, using a second lead approach (FE 231, ADInstruments). Beat‐to‐beat BP was measured via finger photoplethysmography on the left middle finger with a brachial cuff to correct BP values to the level of the heart (Finapres NOVA, Finapres Medical Systems, Amsterdam, The Netherlands). This method has previously been shown to reliably assess the alterations in beat‐to‐beat BP which are well correlated with intra‐arterial recordings and can be used during dCA assessments (Omboni et al., 1993; Sammons et al., 1985). Finally, a mouthpiece and inline gas analyzer (ML 206, ADInstruments) were used to measure the partial pressure of end‐tidal carbon dioxide (P_ET_CO_2_), which were calibrated prior to participant arrival with a known gas concentration (5.00% carbon dioxide_,_ 15.94% oxygen, balance nitrogen) and room air (0.03% carbon dioxide, 20.93% oxygen, and 78.09% nitrogen). During all SSM, participants were coached on their breathing (i.e., breathe deeper, slower, shallower, etc.) to ensure P_ET_CO_2_ values were as comparable between all tasks, as carbon dioxide has been demonstrated to be an important confounding factor to control for when deriving autoregulatory estimates (Willie et al., 2014).

**FIGURE 1:**
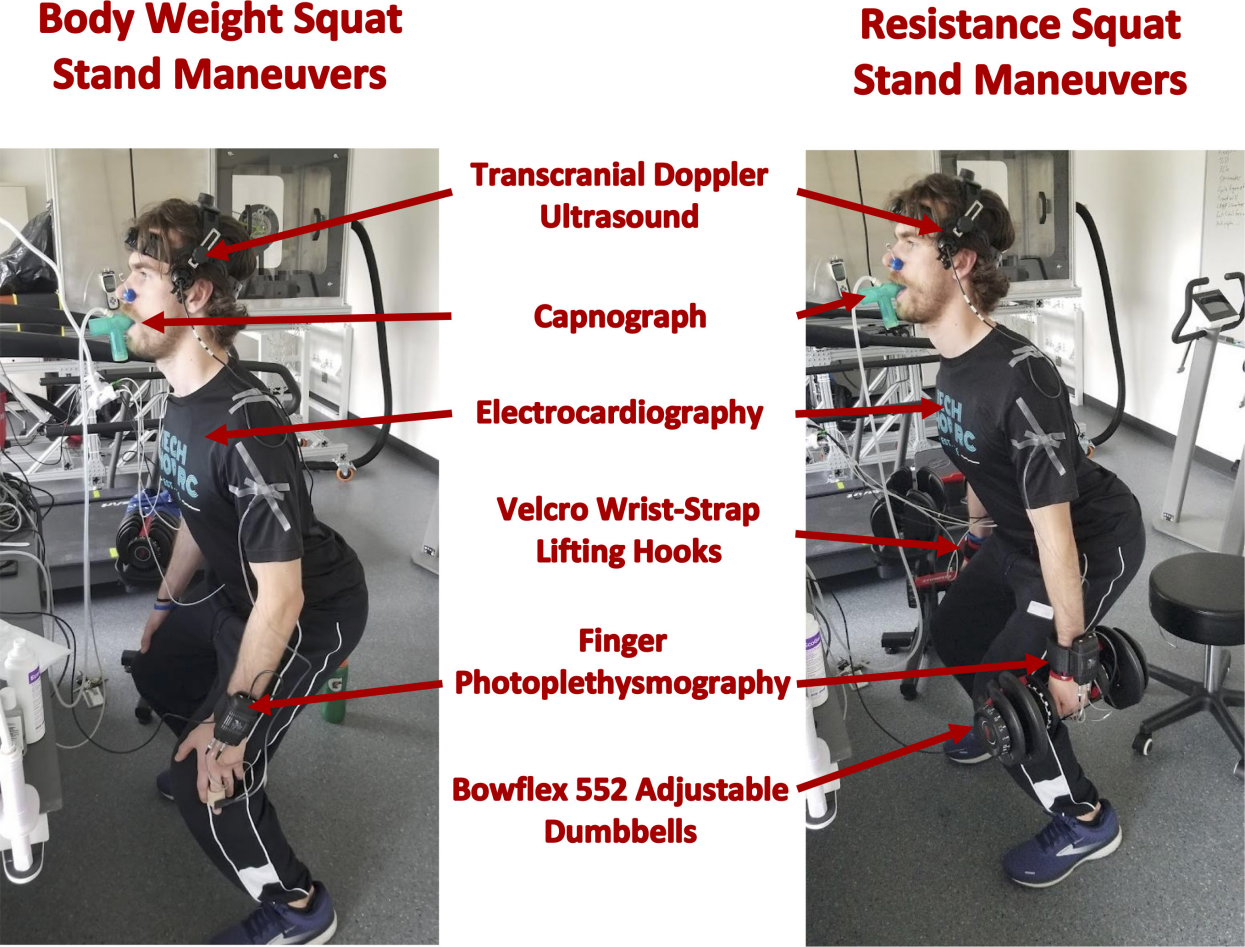
A visual representation of one participant performing the bodyweight squat‐stand maneuvers and the resistance squat‐stand maneuvers with 10% of the individual's bodyweight in each hand for the latter

For the resistance SSM (rSSM), Bowflex 552 Adjustable Dumbbells (Nautilus Inc) were used to provide an additional 10% of an individual's bodyweight in each hand. The weights were selected to the closest 2.5 lbs for each individual based upon body mass. Weights were held in place with padded Velcro wrist‐strap lifting hooks (Iron Bull Strength) to enable the hands to stay relaxed while holding the Bowflex 552 weights, reducing the risk of altering blood flow to the hand. This ensured robust Finapres recordings could concurrently be collected (Figure 1).

### 2.3 Experimental protocol

All data collection occurred during a single laboratory visit at the Cerebrovascular Concussion Laboratory at the University of Calgary. These occurred across an individual's normal workday, which has been shown to have minimal dCA alterations associated with diurnal variation (Burma et al., 2020a). The laboratory conditions were tightly controlled across all sessions for environmental factors: barometric pressure of 668.5 ± 2.3 mmHg; humidity at 48.7 ± 12.2%; and temperature of 21.0 ± 0.3°C. The elevation of the laboratory was 1111 meters above sea level.

Once the equipment was attached, participants completed two, 5‐min bouts of bodyweight SSMs at frequencies of 0.05 and 0.10 Hz. A frequency of 0.05 was chosen as it falls within the very low‐frequency range of autoregulation (0.02–0.07 Hz), which provides an assessment of dCA influences of metabolic, endothelial, neurogenic, and/or myogenic influences (Hamner & Tan, 2014; Hamner et al., 2010; Tan et al., 2013). Likewise, 0.10 Hz was chosen as it falls within the low‐frequency range (0.07–0.20 Hz), accounting for the influence of sympathetic innervation on dCA (Hamner & Tan, 2014; Hamner et al., 2010). Participants completed the SSM in rhythm with a metronome at 6 (0.05 Hz) and 12 beats per minute (0.10 Hz). Following the completion of the bodyweight SSM, participants were allotted an ~30–60 min rest to ensure all physiological variables were comparable to baseline. Following this rest period, participants then completed the same protocol of SSM at randomized frequencies of 0.05 and 0.10 Hz, adding an additional ~10% of bodyweight in each hand (total resistance of 20% bodyweight; Figure 1).

### 2.4 Data processing

Data were sampled at 1000 Hz using commercially available software (Powerlab 16/30 ML880; ADInstruments). Researchers visually inspected all traces and manually corrected all artifacts by interpolating the correct systolic peaks in all traces as required (<0.1% of all traces). The R‐R interval was used to calculate heart rate. Peak‐systolic and end‐diastolic BP were used to calculate MAP from each beat‐to‐beat real‐time BP pulsatile waveform. Similarly, the peak‐systolic and end‐diastolic MCA and PCA velocities were used to calculate mean MCA and PCA velocities, respectively. Calculations of P_ET_CO were done using breath‐to‐breath peak values of partial pressure of carbon dioxide. All data processing was in accordance with the standards put forward by the Cerebral Autoregulation Research Network (CARNet) white paper (Claassen et al., 2016).

### 2.5 Transfer function analysis (TFA) and power spectrum densities

A Welch smoothing method was used to improve spectral estimates, where beat‐to‐beat BP and CBV waveforms were spline interpolated and resampled at 4 Hz (Claassen et al., 2016). As previously described, it is recommended a 300‐s recording is utilized to derive TFA estimates from SSM (Burma et al., 2021). Data from the 300‐s recording were detrended and passed through a Hanning window (five windows, 50% overlap, 100‐s per window). Cross‐spectrums were taken between BP and CBV at each phase of the cardiac cycle (e.g., diastolic arterial pressure and diastolic blood velocity). For each cardiac cycle component, the values were then divided by the respective autospectrum. Autoregulatory TFA estimates were then computed at the driven point‐estimates of 0.05 and 0.10 Hz for the diastolic, mean, and systolic aspects of the cardiac cycle (Burma et al., 2020a; Burma, Copeland, Macaulay, Khatra, Wright, et al., 2020; Smirl et al., 2018). The outcome measures of interest included TFA power spectrum densities (PSD), coherence, phase, gain, and normalized gain. The power spectrum density quantifies the power/strength of a signal within the frequency domain (Zhang et al., 1998). Coherence describes the input‐output relationship of the cerebral pressure‐flow relationship on an arbitrary scale ranging from 0 (no relationship) to 1 (complete relationship; Zhang et al., 1998). Phase describes the temporal alignment (radians), whereas absolute gain (cm/s/mmHg) quantifies the amplitude modulation within the cerebral pressure‐flow relationship (Zhang et al., 1998). Normalized gain (%/mmHg) was reported in addition to absolute gain, as it has shown to be more reliable (Burma et al., 2020a; Smirl et al., 1985). Given the TFA computation method used (i.e., spectral smoothing, 50% overlap, and five windows), the critical value for coherence was determined in alignment with the *CARNet White Paper* at an alpha value of 0.01 (Claassen et al., 2016). Thus the a priori coherence threshold was set at 0.46. Phase wraparound was not present in any of the data samples.

### 2.6 Sample size calculation

An a priori sample size calculation was conducted with G*power (v3.1.9.6) using the studies by Labrecque, Rahimaly, et al. (Labrecque, Rahimaly, et al., 2019) and Favre and Serrador (2019) which found differences in TFA metrics between males and females. These studies had sample sizes of 11 and 13 males and females, respectively. The aforementioned studies found significant differences between chromosomal sexes for MAP PSD (Favre & Serrador, 2019), MCA PSD (Favre & Serrador, 2019), coherence (Favre & Serrador, 2019; Labrecque, Rahimaly, et al., 2019), phase (Favre & Serrador, 2019), gain (Labrecque, Rahimaly, et al., 2019), and normalized gain (Favre & Serrador, 2019). From these comparisons, the calculated *f* effect size was 0.65, which corresponds to an eta squared effect size of 0.297 [large]. Nevertheless, to ensure adequate statistical power, the effect size was lowered to 0.26, which is the threshold between a moderate and large eta squared effect size (Bakeman, 2005). As no research has been conducted into the domain of resistance exercise with SSM to assess dCA *during* the rSSM, the sample size required for the sex comparisons was hypothesized to be consistent with the bodyweight SSM and rSSM comparisons. An important consideration is a fact the comparison between sex has a greater likelihood of being influenced by covariates, as different people are being compared (Mills et al., 2009). Conversely, for the within‐group comparison (type of SSM), the individuals would act as their own controls and therefore all covariates within individuals would be the same (Maclure, 1991). Henceforth, with the same number of subjects, a greater power would be elicited for the within‐group comparison as opposed to the between‐group comparison (Jager et al., 2008; Maclure, 1991). Therefore, the eta squared effect size of 0.26 was used for both the between‐group (sex) and within‐group comparisons (type of SSM). To ensure the within‐between interaction was adequately powered, the eta squared was divided in half (0.13). Conclusively, it was found that with a power of 80%, an alpha of 0.05, two tails, two groups, and two measures; a total sample size of 10, 20, and 24 participants were required to power the within‐group, between‐group, and within‐between interaction comparisons, respectively. Therefore, the present study required 12 female and 12 male individuals to address the overall aims of the paper.

### 2.7 Statistical analysis

All statistical analyses were completed in RStudio (v1.4.1060). The assumptions of linear mixed effect models were run to determine if these could be used to quantify the impact of added resistance on the cerebral pressure‐flow relationship. Type of squat (bodyweight or resistance) was defined as a repeated measure, whereas sex was a fixed effect. While previous research has highlighted unbiased estimates can be produced from linear mixed effect models with violated assumptions, these should nonetheless be considered on a case‐by‐case basis to avoid highly imprecise interpretations (Schielzeth et al., 2020). Conversely, F‐Tests (i.e., ANOVA) have demonstrated to be robust in mitigating against Type I errors in the presence of non‐normal data and violated assumptions when running basic models (e.g., one‐way models, two factorial models, etc.; Blanca et al., 2017). Therefore, the latter analyses were used in the current investigation. Levene's tests were used to quantify the homogeneity of variances assumption, which if this assumption was violated, the data for a given comparison were log‐transformed. Two‐by‐four one‐way ANOVA models were used to examine potential physiological differences between all four SSM performed by chromosomal sex. Two‐factorial two‐by‐two repeated measures ANOVA models were used to compare differences in tasks (bodyweight vs. rSSM) and sex (female vs. male). Tukey's honestly significant difference pairwise comparisons were performed to determine differences in the presence of a significant omnibus test from the 2 × 4 and 2 × 2 ANOVA. Generalized eta squared ($\eta_{G}^{2}$) and Cohen's *d* effect sizes were calculated as previous research has questioned the utility of basing inferences on *p*‐values independently (Amrhein et al., 2019; Panagiotakos, 2008). Therefore, all inferences from the current manuscript were based upon an a priori alpha of 0.05 (Thiese et al., 2016), as well as the effect size value (Amrhein et al., 2019; Panagiotakos, 2008). The $\eta_{G}^{2}$coefficients were determined with threshold of <0.02 (negligible), 0.02–0.13 (small), 0.13–0.26 (moderate), and >0.26 (large; Bakeman, 2005). Negligible, small, moderate, and large Cohen's *d* effect sizes are quantified by thresholds of <0.2, 0.2–0.5, 0.5–0.8, and >0.8, respectively (Lakens, 2013). To determine the reliability of physiological variables across the four tasks, coefficient of variation (CoV) values were computed as the quotient of the standard deviation over the average value and displayed in a percentage (Atkinson & Nevill, 1998). A bootstrap analysis with 10,000 resamples was then conducted on the calculated quotient for all subjects, which produced the CoV 95% CIs. Data are presented as mean ± SD or mean ± 95% CIs.

## 3 RESULTS

### 3.1 Data for each comparison

Given the complications of concurrent measuring beat‐to‐beat BP, while holding 10% of one's body weight in each arm, some data were not capable of being utilized. Therefore, to ensure poor data would not impact the results, data were excluded if the Finapres trace did not produce >4‐min of quality data. To ensure transparency, the following number of females (F)/males (M) were used for the following comparisons: 0.05 Hz diastole (12F/10 M), 0.05 Hz mean (12F/11 M), 0.05 Hz systole (12F/11 M), 0.10 Hz diastole (11F/12 M), 0.10 Hz mean (12F/12 M), and 0.10 Hz systole (11F/11 M).

### 3.2 Physiological metrics

All cardiovascular, cerebrovascular, and respiratory variables during all SSM are displayed in Table 1. Across the four SSM, a sex main effect was found for MCA velocity (*F*_1,92_ = 40.1; *p* < 0.001; $\eta_{G}^{2}$ = 0.30 [large]), PCA velocity (*F*_1,92_ = 11.7; *p* < 0.001; $\eta_{G}^{2}$ = 0.11 [small]), systolic BP (*F*_1,92_ = 7.10; *p* = 0.009; $\eta_{G}^{2}$ = 0.07 [small]), and heart rate (*F*_1,92_ = 6.17; *p* = 0.012; $\eta_{G}^{2}$ = 0.06 [small]; Table 1). A type of squat main effect was found for diastolic BP (*F*_3,92_ = 12.3; *p* < 0.001; $\eta_{G}^{2}$ = 0.29 [moderate]) and MAP (*F*_3,92_ = 11.1; *p* < 0.001; $\eta_{G}^{2}$ = 0.29 [moderate]). No other type, sex, or type by sex main effect were present for all physiological variables (*F*_3,92_ < 3.88; *p* = 0.052; $\eta_{G}^{2}$ < 0.04 [negligible/small]; Table 1). Furthermore, Table 1 highlights the CoV for all metrics between tasks and the associated 95% CI. Of note, the greatest variability was seen between the SSM for diastolic BP and MAP (Table 1).

**TABLE 1:** Respiratory, cardiovascular, and cerebrovascular variables during body weight squat‐stand maneuvers (SSM) and resistance squat‐stand maneuvers (rSSM) in 25 individuals (13 females/12 males)

	0.05 Hz SSM	0.10 Hz SSM	0.05 Hz rSSM	0.10 Hz rSSM	CoV statistics
P_ET_CO_2_ (mmHg)	39.1 ± 3.4	39.9 ± 3.7	39.2 ± 3.1	39.7 ± 3.8	4.5% (3.5%–5.5%)
Female	38.9 ± 3.3	39.0 ± 3.2	38.6 ± 2.9	39.5 ± 4.0	4.9% (3.5%–6.3%)
Male	39.3 ± 3.7	40.9 ± 4.0	40.0 ± 3.3	40.0 ± 3.8	4.0% (2.7%–5.4%)
Respiratory rate (BPM)^a^	16.6 ± 4.3	16.3 ± 4.5	17.3 ± 4.5	17.5 ± 5.1	10.2% (8.3%–12.1%)
Female	16.2 ± 4.7	16.2 ± 5.4	16.9 ± 5.0	17.2 ± 5.9	10.1% (7.3%–12.8%)
Male	17.0 ± 3.9	16.4 ± 4.5	17.7 ± 4.1	17.8 ± 4.2	10.4% (7.9%–12.9%)
Mean arterial pressure (mmHg)	81.9 ± 11.1	81.9 ± 11.5	96.6 ± 12.1	94.4 ± 12.1	12.9% (10.6%–15.1%)
Female	82.9 ± 14.7	83.2 ± 14.3	98.8 ± 15.3	94.5 ± 13.8	14.6% (11.1%–18.0%)
Male	80.8 ± 5.50	80.5 ± 8.0	94.3 ± 7.3	94.3 ± 10.7	11.0% (8.5%–13.4%)
MCAv (cm/s)	59.0 ± 15.9	59.8 ± 14.6	61.4 ± 17.2	61.7 ± 15.1	7.7% (6.2%–9.2%)
Female	68.0 ± 16.3	68.1 ± 13.9	68.7 ± 16.1	69.6 ± 19.2	9.2% (6.8%–11.5%)
Male	49.3 ± 8.0	50.7 ± 9.0	52.5 ± 8.8	54.0 ± 9.5	6.1% (4.6%–7.6%)
PCAv (cm/s)	36.6 ± 8.8	36.7 ± 8.5	37.6 ± 9.7	37.9 ± 8.8	7.6% (5.5%–9.7%)
Female	39.8 ± 10.5	39.4 ± 9.6	40.3 ± 11.0	40.6 ± 9.4	7.3% (4.3%–10.6%)
Male	33.2 ± 4.9	33.7 ± 6.4	34.7 ± 7.3	35.0 ± 7.5	7.8% (5.2%–10.5%)
Heart rate (bpm)	87.9 ± 15.7	87.8 ± 18.4	86.1 ± 20.4	86.0 ± 24.6	8.0% (5.3%–10.6%)
Female	93.5 ± 18.7	93.2 ± 22.8	90.9 ± 26.1	89.1 ± 31.4	9.5% (4.9%–14.1%)
Male	81.9 ± 8.7	82.0 ± 10.0	80.8 ± 10.6	82.6 ± 15.1	6.3% (4.1%–8.4%)
Systolic arterial pressure (mmHg)	129.9 ± 16.9	132.0 ± 17.5	139.4 ± 20.0	139.7 ± 19.6	8.1% (6.4%–9.8%)
Female	125.4 ± 21.0	127.8 ± 20.8	136.2 ± 10.0	132.9 ± 17.6	10.0% (7.4%–12.7%)
Male	134.7 ± 9.70	136.6 ± 12.4	142.9 ± 14.4	147.0 ± 19.7	6.1% (4.3%–7.9%)
Diastolic arterial pressure (mmHg)	61.7 ± 10.3	60.9 ± 11.6	76.3 ± 11.1	72.9 ± 11.6	16.7% (13.9%–19.5%)
Female	63.4 ± 13.2	62.7 ± 13.4	79.5 ± 13.4	74.7 ± 13.5	19.4% (15.2%–23.6%)
Male	59.8 ± 5.60	59.0 ± 9.3	72.9 ± 7.1	71.0 ± 9.4	13.7% (12.3%–17.1%)

Note

Values are mean ± SD for group averaged (top line), female (middle line), and male (bottom line) data. It is important to note while various aspects of the cerebral pressure‐flow relationship were removed due to technical difficulties collecting data with the resistance weights, some data were salvageable from all participants. Therefore, the presented data are averaged from all participants.

Abbreviations: bpm, beats per minute);BPM, breaths per minute; mmHg, millimeters of mercury; P_ET_CO_2,_ End tidal values of carbon dioxide; RR, respiratory rate.

^a^ Respiratory rate (*p* < 0.001; Cohen's *d* = 0.81) differed between sexes. However, it should be noted this did not impact the P_ET_CO_2_ values between sexes, ensuring both females and males were at a similar state of eucapnia. The coefficient of variation (CoV) values were calculated using the mean values from each subject, where a bootstrap approach with 10,000 resamples.

### 3.3 Power spectrum density task, sex, and task by sex main effects and pairwise comparisons

The MAP, MCA, and PCA PSD task, sex, and task by sex main effects are displayed in Table 2 for all aspects of the cardiac cycle at both 0.05 and 0.10 Hz. When taking both *p*‐values and effect sizes into consideration, the BP PSD at 0.05 Hz displayed a significant sex omnibus test at diastole (*F*_1,41_ = 5.50; *p* = 0.048; $\eta_{G}^{2}$ = 0.13 [moderate]), mean (*F*_1,42_ = 13.7; *p* < 0.001; $\eta_{G}^{2}$ = 0.25 [moderate]), and systole (*F*_1,41_ = 14.2; *p* = 0.001; $\eta_{G}^{2}$ = 0.25 [moderate]; Table 2). Whereas the type and type by sex main effects for the BP PSD at 0.05 Hz were not significant (all *p* > 0.102) and had negligible or small effect sizes (all $\eta_{G}^{2}$ < 0.09; Table 2). At 0.10 Hz, all mean BP PSD main effects were significant, albeit with some having small effect sizes: task (*F*_1,44_ = 8.37; *p* = 0.024; $\eta_{G}^{2}$ = 0.16 [moderate]), sex (*F*_1,44_ = 5.62; *p* = 0.044; $\eta_{G}^{2}$ = 0.11 [small]), and task by sex (*F*_1,44_ = 5.79; *p* = 0.040; $\eta_{G}^{2}$ = 0.12 [small]; Table 2). Additionally, for BP PSD at 0.10 Hz, diastole task by sex (*F*_1,43_ = 6.64; *p* = 0.026; $\eta_{G}^{2}$ = 0.12 [small]) and systole task (*F*_1,42_ = 5.45; *p* = 0.048; $\eta_{G}^{2}$ = 0.12 [small]) omnibus tests were significant (Table 2). All other BP PSD at 0.10 Hz were not significant (all *p* > 0.062) and had negligible/small effect sizes (all $\eta_{G}^{2}$ < 0.11; Table 2). Finally, across the cardiac cycle at both frequencies, all MCA and PCA PSD main effects were not significant (all *p* > 0.058) and had a negligible or small effect size (all $\eta_{G}^{2}$ < 0.11; Table 2).

**TABLE 2:** Blood pressure (BP), Middle cerebral artery (MCA), and Posterior cerebral artery (PCA) Power spectrum densities (PSD) across the cardiac cycle during squat stand maneuvers (SSM) at 0.05 and 0.10 Hz in 25 individuals (13 females/12 males)

	Body weight SSM	Resistance SSM	Effect type	Test statistics
*0.05 Hz*				
Dia BP PSD				
Female	13,852 ± 15,170	9026 ± 4867	Task effect	*F*_1,41_ = 0.01; *p* = 0.997; $\eta_{G}^{2}$ = 0.01
Male	16,258 ± 8526	21,494 ± 10,235	Sex effect	*F*_1,41_ = 5.50; *p* = 0.048; $\eta_{G}^{2}$ = 0.13
Total	15,003 ± 12,232	14,693 ± 9884	Interaction effect	*F*_1,41_ = 2.60; *p* = 0.224; $\eta_{G}^{2}$ = 0.06
Dia MCA PSD				
Female	10,658 ± 11,193	7163 ± 5619	Task effect	*F*_1,41_ = 0.35; *p* = 0.920; $\eta_{G}^{2}$ = 0.01
Male	15,027 ± 12,582	15,052 ± 11,787	Sex effect	*F*_1,41_ = 3.73; *p* = 0.120; $\eta_{G}^{2}$ = 0.08
Total	12,748 ± 11,814	10,749 ± 9605	Interaction effect	*F*_1,41_ = 0.31; *p* = 0.935; $\eta_{G}^{2}$ = 0.01
Dia PCA PSD				
Female	7138 ± 7398	5224 ± 4577	Task effect	*F*_1,41_ = 0.36; *p* = 0.918 $\eta_{G}^{2}$ = 0.01
Male	8864 ± 6115	8796 ± 4696	Sex effect	*F*_1,41_ = 2.25; *p* = 0.242; $\eta_{G}^{2}$ = 0.05
Total	7963 ± 6719	6848 ± 4872	Interaction effect	*F*_1,41_ = 0.28; *p* = 0.948; $\eta_{G}^{2}$ = 0.01
Mean BP PSD				
Female	15,689 ± 10,723	15,792 ± 8929	Task effect	*F*_1,42_ = 3.34; *p* = 0.150; $\eta_{G}^{2}$ = 0.07
Male	23,197 ± 12,824	38,892 ± 21,181	Sex effect	*F*_1,42_ = 13.7; *p* < 0.001; $\eta_{G}^{2}$ = 0.25
Total	19,280 ± 12,122	26,840 ± 19,570	Interaction effect	*F*_1,42_ = 3.54; *p* = 0.134; $\eta_{G}^{2}$ = 0.08
Mean MCA PSD				
Female	8559 ± 6798	6560 ± 5275	Task effect	*F*_1,42_ = 0.16; *p* = 0.978; $\eta_{G}^{2}$ = 0.01
Male	9677 ± 7237	13,751 ± 10,550	Sex effect	*F*_1,42_ = 3.40; *p* = 0.134; $\eta_{G}^{2}$ = 0.08
Total	9094 ± 6873	9999 ± 8831	Interaction effect	*F*_1,42_ = 1.81; *p* = 0.370; $\eta_{G}^{2}$ = 0.04
Mean PCA PSD				
Female	4186 ± 3950	3434 ± 2628	Task effect	*F*_1,42_ = 0.16; *p* = 0.978; $\eta_{G}^{2}$ = 0.01
Male	5089 ± 3894	6873 ± 4895	Sex effect	*F*_1,42_ = 3.56; *p* = 0.122; $\eta_{G}^{2}$ = 0.08
Total	4618 ± 3861	5079 ± 4175	Interaction effect	*F*_1,42_ = 1.21; *p* = 0.544; $\eta_{G}^{2}$ = 0.03
Sys BP PSD				
Female	22,114 ± 18,953	26,919 ± 17,464	Task effect	*F*_1,41_ = 3.85; *p* = 0.102; $\eta_{G}^{2}$ = 0.08
Male	49,124 ± 32,398	96,395 ± 78,369	Sex effect	*F*_1,41_ = 14.2; *p* = 0.001; $\eta_{G}^{2}$ = 0.25
Total	35,031 ± 29,053	60,147 ± 64,834	Interaction effect	*F*_1,41_ = 2.75; *p* = 0.210 $\eta_{G}^{2}$ = 0.06
Sys MCA PSD				
Female	4289 ± 3434	4967 ± 3427	Task effect	*F*_1,41_ = 5.11; *p* = 0.058; $\eta_{G}^{2}$ = 0.11
Male	4023 ± 2539	9612 ± 7376	Sex effect	*F*_1,41_ = 2.46; *p* = 0.248; $\eta_{G}^{2}$ = 0.06
Total	4161 ± 2974	7289 ± 6095	Interaction effect	*F*_1,41_ = 3.25; *p* = 0.158; $\eta_{G}^{2}$ = 0.07
Sys PCA PSD				
Female	1550 ± 2474	2113 ± 2495	Task effect	*F*_1,41_ = 3.66; *p* = 0.126; $\eta_{G}^{2}$ = 0.08
Male	1726 ± 1475	4040 ± 3013	Sex effect	*F*_1,41_ = 2.03; *p* = 0.324; $\eta_{G}^{2}$ = 0.05
Total	1634 ± 2012	2989 ± 2859	Interaction effect	*F*_1,41_ = 1.47; *p* = 0.464; $\eta_{G}^{2}$ = 0.04
*0.10 Hz*				
Dia BP PSD				
Female	16,490 ± 13,257	13,792 ± 7490	Task effect	*F*_1,43_ = 2.99; *p* = 0.182; $\eta_{G}^{2}$ = 0.07
Male	14,423 ± 4889	27,066 ± 12,479	Sex effect	*F*_1,43_ = 3.33; *p* = 0.150; $\eta_{G}^{2}$ = 0.07
Total	15,456 ± 9829	20,718 ± 12,220	Interaction effect	*F*_1,43_ = 6.64; *p* = 0.026; $\eta_{G}^{2}$ = 0.12
Dia MCA PSD				
Female	30,657 ± 28,600	33,125 ± 22,721	Task effect	*F*_1,43_ = 1.12; *p* = 0.592; $\eta_{G}^{2}$ = 0.03
Male	22,846 ± 15,314	34,471 ± 23,897	Sex effect	*F*_1,43_ = 0.24; *p* = 0.956; $\eta_{G}^{2}$ = 0.01
Total	26,752 ± 22,787	33,827 ± 22,818	Interaction effect	*F*_1,43_ = 0.46; *p* = 0.684; $\eta_{G}^{2}$ = 0.01
Dia PCA PSD				
Female	17,063 ± 13,265	17,726 ± 11,052	Task effect	*F*_1,43_ = 1.11; *p* = 0.596; $\eta_{G}^{2}$ = 0.03
Male	13,617 ± 8607	19,543 ± 10,177	Sex effect	*F*_1,43_ = 0.08; *p* = 0.992; $\eta_{G}^{2}$ = 0.01
Total	15,340 ± 11,076	18,674 ± 10,400	Interaction effect	*F*_1,43_ = 0.68; *p* = 0.813; $\eta_{G}^{2}$ = 0.02
Mean BP PSD				
Female	19,480 ± 10,616	21,162 ± 8694	Task effect	*F*_1,44_ = 8.37; *p* = 0.012; $\eta_{G}^{2}$ = 0.16
Male	19,363 ± 8149	37,608 ± 17,729	Sex effect	*F*_1,44_ = 5.62; *p* = 0.044; $\eta_{G}^{2}$ = 0.11
Total	19,421 ± 9256	29,385 ± 16,032	Interaction effect	*F*_1,44_ = 5.79; *p* = 0.040; $\eta_{G}^{2}$ = 0.12
Mean MCA PSD				
Female	27,512 ± 27,000	30,591 ± 21,013	Task effect	*F*_1,44_ = 2.13; *p* = 0.352; $\eta_{G}^{2}$ = 0.05
Male	15,499 ± 10,745	29,473 ± 18,724	Sex effect	*F*_1,44_ =1.26; *p* = 0.534; $\eta_{G}^{2}$ =0.03
Total	21,506 ± 21,012	30,032 ± 19,472	Interaction effect	*F*_1,44_ = 0.87; *p* = 0.712; $\eta_{G}^{2}$ = 0.02
Mean PCA PSD				
Female	11,247 ± 10,978	13,335 ± 10,640	Task effect	*F*_1,44_ = 1.89; *p* = 0.354; $\eta_{G}^{2}$ = 0.04
Male	8205 ± 6458	13,577 ± 8869	Sex effect	*F*_1,44_ = 0.27; *p* = 0.948; $\eta_{G}^{2}$ = 0.01
Total	9726 ± 8944	13,456 ± 9580	Interaction effect	*F*_1,44_ = 0.37; *p* = 0.912; $\eta_{G}^{2}$ = 0.01
Sys BP PSD				
Female	22,470 ± 21,981	30,234 ± 13,354	Task effect	*F*_1,42_ = 5.45; *p* = 0.048; $\eta_{G}^{2}$ = 0.12
Male	29,854 ± 20,670	62,775 ± 49,826	Sex effect	*F*_1,42_ = 4.97; *p* = 0.062; $\eta_{G}^{2}$ = 0.11
Total	26,162 ± 21,205	46,504 ± 39,300	Interaction effect	*F*_1,42_ = 2.08; *p* = 0.312; $\eta_{G}^{2}$ = 0.05
Sys MCA PSD				
Female	13,935 ± 13,652	18,862 ± 16,180	Task effect	*F*_1,42_ = 3.22; *p* = 0.160; $\eta_{G}^{2}$ = 0.07
Male	5015 ± 4568	13,847 ± 13,698	Sex effect	*F*_1,42_ = 3.39; *p* = 0.146; $\eta_{G}^{2}$ = 0.08
Total	9475 ± 11,551	16,355 ± 14,853	Interaction effect	*F*_1,42_ = 0.26; *p* = 0.952; $\eta_{G}^{2}$ = 0.01
Sys PCA PSD				
Female	5666 ± 8081	10,157 ± 8420	Task effect	*F*_1,42_ = 4.25; *p* = 0.090; $\eta_{G}^{2}$ = 0.09
Male	2088 ± 2233	5334 ± 5065	Sex effect	*F*_1,42_ = 5.02; *p* = 0.060; $\eta_{G}^{2}$ = 0.10
Total	3877 ± 6080	7640 ± 7150	Interaction effect	*F*_1,42_ = 0.11; *p* = 0.989; $\eta_{G}^{2}$ = 0.01

Note

Data are displayed as mean ± SD for males, females, and the total of both combined. The test statistics were determined through a 2 × 2 Analysis of Variance to determine the main effects of type of SSM and sex. Post‐hoc comparisons were determined through Tukey's honestly significant difference. Effect sizes were determined through generalized eta squared ($\eta_{G}^{2}$), with thresholds of <0.02 (negligible), 0.02–0.13 (small), 0.13–0.26 (moderate), and >0.26 (large).

The post‐hoc comparisons revealed males had a greater BP PSD at 0.05 Hz in diastole (*p* = 0.024; Cohen's *d* = 0.70 [moderate]), mean (*p* < 0.001; Cohen's *d* = 1.02 [large]), and systole (*p* < 0.001; Cohen's *d* = 1.04 [large]; Table 2). At 0.10 Hz, the diastolic BP PSD time by sex interaction revealed differences where male rSSM were greater than: (Ainslie & Hoiland, 1985) Female bodyweight SSM (*p* = 0.034; Cohen's *d* = 0.83 [large]), (2) female rSSM (*p* = 0.017; Cohen's *d* = 1.29 [large]), and (3) male bodyweight SSM (*p* = 0.020; Cohen's *d* = 1.33 [large]). No other comparisons were significant (all *p* > 0.921; Cohen's *d *< 0.25 [small]) in this interaction effect. Furthermore, mean BP PSD at 0.10 Hz was greater during rSSM compared to bodyweight SSM (*p* = 0.006; Cohen's *d* = 0.76 [moderate]) and within males compared to females (*p* = 0.022; Cohen's *d* = 0.61 [moderate]). The type by sex interaction indicated male rSSM were greater than: (1) Female bodyweight SSM (*p* = 0.003; Cohen's *d* = 1.24 [large]), (2) female rSSM (*p* = 0.008; Cohen's *d* = 1.18 [large]), and (3) male bodyweight SSM (*p* = 0.003; Cohen's *d* = 1.32 [large]); however, no other groups were different (all *p* > 0.983; Cohen's *d *< 0.21 [small]). Finally, rSSM elicited a greater systolic BP PSD at 0.10 Hz compared to bodyweight SSM (*p* = 0.025; Cohen's *d* = 0.64 [moderate]).

### 3.4 Dynamic cerebral autoregulation main effects

**FIGURE 2:**
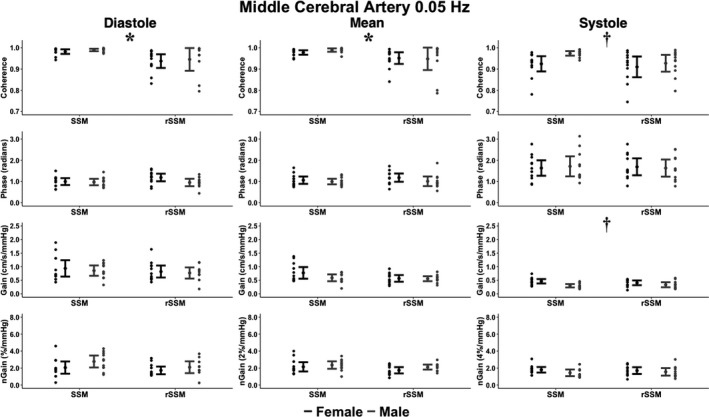
The cerebral pressure‐flow relationship expressed through transfer function analysis metrics within the middle cerebral artery during bodyweight squat stand maneuvers (SSM) and resistance SSM (rSSM) at 0.05 Hz across the cardiac cycle. The following total number of participants (T), females (F), and males (M) were included for the comparisons within each phase of the cardiac cycle: diastole (22T/12F/10 M), mean (23T/12F/11 M), and systole (23T/12F/11 M). It is important to note the normalized gain (nGain) metrics were doubled and sextupled within the mean and systolic components of the cardiac cycle, respectively. This was done to provide a visual representation of the differences between metrics only, where all statistical analysis and interpretations were drawn from the raw data. Data are presented as mean ± 95% CIs. Two‐by‐two Analysis of Variance with generalized eta squared effect sizes were used to determine groups effects of type of squat, sex, and type‐by‐sex interaction. Post‐hoc comparisons were performed using Tukey's honestly significant difference pairwise comparisons with Cohen's *d*effect sizes. The asterisks (*) denotes where a task main effect difference was noted between SSM and rSSM, whereas the dagger (†) denotes where a sex main effect difference was noted between males and females. Centimeters (cm), millimeters of mercury (mmHg), and seconds (s)

**FIGURE 3:**
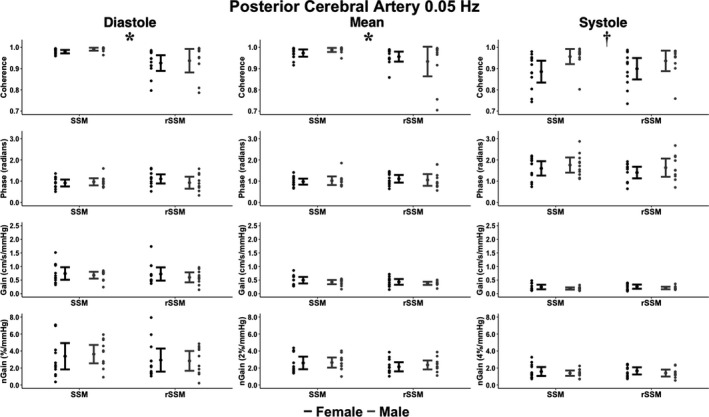
The cerebral pressure‐flow relationship expressed through transfer function analysis metrics within the posterior cerebral artery during bodyweight squat stand maneuvers (SSM) and resistance SSM (rSSM) at 0.05 Hz across the cardiac cycle. The following total number of participants (T), females (F), and males (M) were included for the comparisons within each phase of the cardiac cycle: Diastole (23T/11F/12 M), mean (24T/12F/12 M), and systole (22T/11F/11 M). It is important to note the normalized gain (nGain) metrics were doubled and sextupled within the mean and systolic components of the cardiac cycle, respectively. This was done to provide a visual representation of the differences between metrics only, where all statistical analysis and interpretations were drawn from the raw data. Data are presented as mean ± 95% CIs. Two‐by‐two Analysis of Variance with generalized eta squared effect sizes were used to determine groups effects of type of squat, sex, and type‐by‐sex interaction. Post‐hoc comparisons were performed using Tukey's honestly significant difference pairwise comparisons with Cohen's *d*effect sizes. The asterisks (*) denotes where a task main effect difference was noted between SSM and rSSM, whereas the dagger (†) denotes where a sex main effect difference was noted between males and females. Centimeters (cm), millimeters of mercury (mmHg), and seconds (s)

**FIGURE 4:**
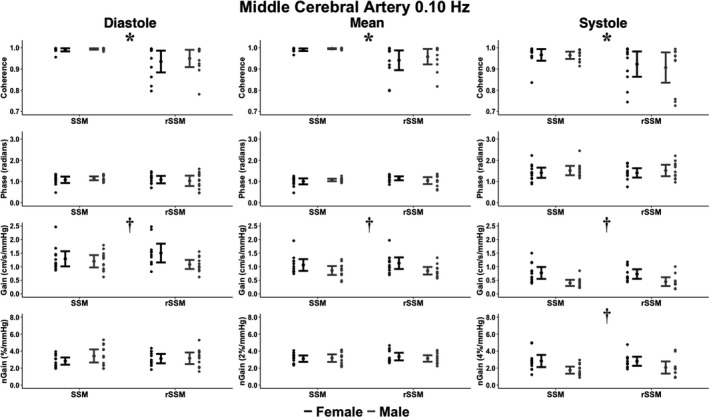
The cerebral pressure‐flow relationship expressed through transfer function analysis metrics within the middle cerebral artery during bodyweight squat stand maneuvers (SSM) and resistance SSM (rSSM) at 0.10 Hz across the cardiac cycle. The following total number of participants (T), females (F), and males (M) were included for the comparisons within each phase of the cardiac cycle: Diastole (22T/12F/10 M), mean (23T/12F/11 M), and systole (23T/12F/11 M). It is important to note the normalized gain (nGain) metrics were doubled and sextupled within the mean and systolic components of the cardiac cycle, respectively. This was done to provide a visual representation of the differences between metrics only, where all statistical analysis and interpretations were drawn from the raw data. Data are presented as mean ±95% CIs. Two‐by‐two Analysis of Variance with generalized eta squared effect sizes were used to determine groups effects of type of squat, sex, and type‐by‐sex interaction. Post‐hoc comparisons were performed using Tukey's honestly significant difference pairwise comparisons with Cohen's *d*effect sizes. The asterisks (*) denotes where a task main effect difference was noted between SSM and rSSM, whereas the dagger (†) denotes where a sex main effect difference was noted between males and females. Centimeters (cm), millimeters of mercury (mmHg), and seconds (s)

**FIGURE 5:**
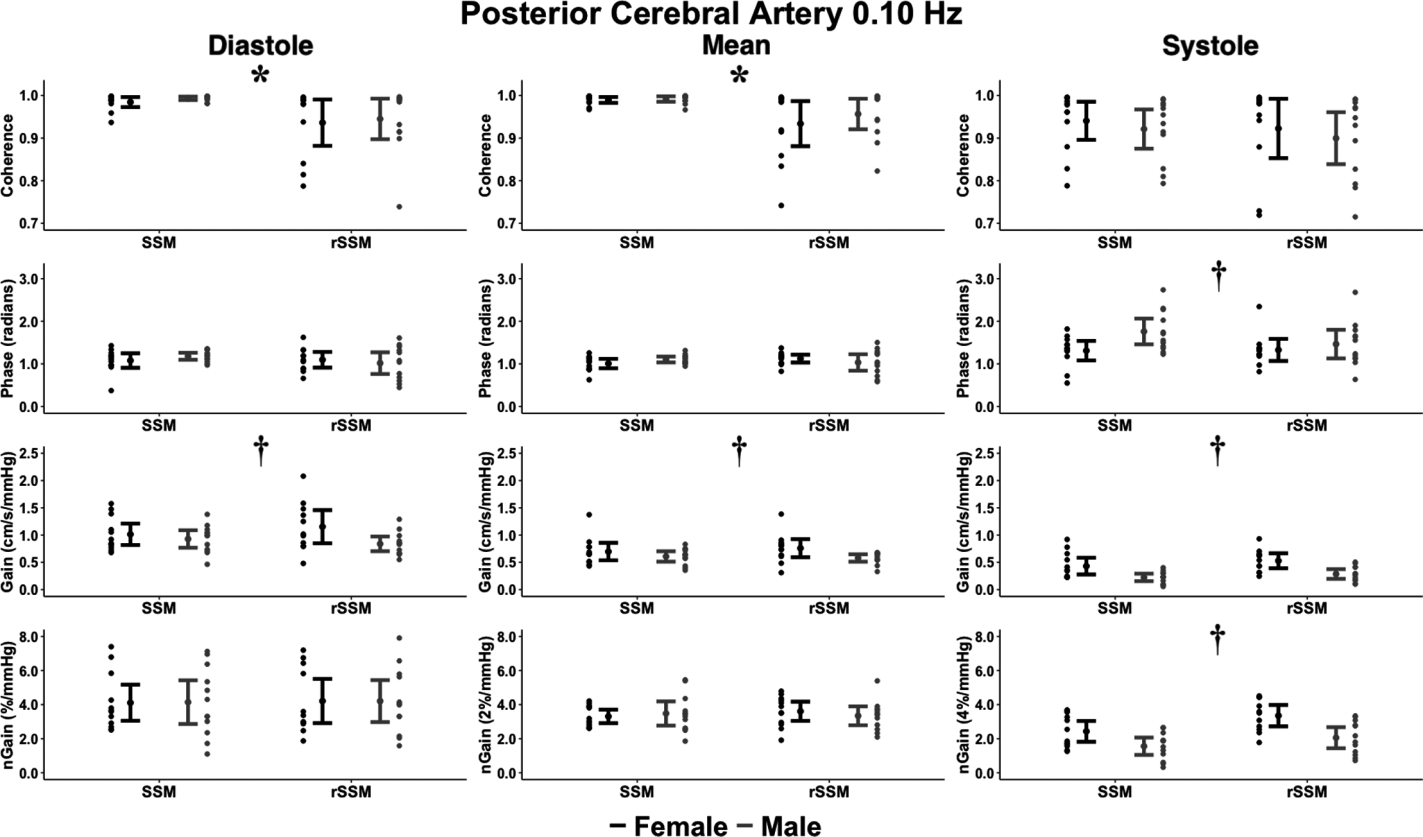
The cerebral pressure‐flow relationship expressed through transfer function analysis metrics within the posterior cerebral artery during bodyweight squat stand maneuvers (SSM) and resistance SSM (rSSM) at 0.10 Hz across the cardiac cycle. The following total number of participants (T), females (F), and males (M) were included for the comparisons within each phase of the cardiac cycle: diastole (23T/11F/12M), mean (24T/12F/12M), and systole (22T/11F/11M). It is important to note the normalized gain (nGain) metrics were doubled and sextupled within the mean and systolic components of the cardiac cycle, respectively. This was done to provide a visual representation of the differences between metrics only, where all statistical analysis and interpretations were drawn from the raw data. Data are presented as mean ± 95% CIs. Two‐by‐two Analysis of Variance with generalized eta squared effect sizes were used to determine groups effects of type of squat, sex, and type‐by‐sex interaction. Post‐hoc comparisons were performed using Tukey's honestly significant difference pairwise comparisons with Cohen's *d* effect sizes. The asterisks (*) denotes where a task main effect difference was noted between SSM and rSSM, whereas the dagger (†) denotes where a sex main effect difference was noted between males and females. Centimeters (cm), millimeters of mercury (mmHg), and seconds (s)

A main effect for type of SSM was present at 0.05 for MCA diastolic coherence (*F*_1,41_ = 6.42; *p *= 0.015; $\eta_{G}^{2}$ = 0.14 [small]), MCA mean coherence (*F*_1,42_ = 4.11; *p* = 0.049; $\eta_{G}^{2}$ = 0.09 [small]), PCA diastolic coherence (*F*_1,41_ = 7.85; *p* = 0.008; $\eta_{G}^{2}$ = 0.16 [small]), and PCA mean coherence (*F*_1,42_ = 4.28; *p* = 0.045; $\eta_{G}^{2}$ = 0.09 [small]; Figures 2 and 3). Further, at 0.05 Hz, a sex main effect was present for MCA systolic coherence (*F*_1,41_ = 4.28; *p* = 0.045; $\eta_{G}^{2}$ = 0.10 [small]), MCA systolic gain (*F*_1,41_ = 10.4; *p* = 0.003; $\eta_{G}^{2}$ = 0.20 [small]), and PCA systolic coherence (*F*_1,41_ = 6.13; *p* = 0.018; $\eta_{G}^{2}$ = 0.13 [moderate]; Figures 2 and 3). At 0.10 Hz, type of SSM main effects were present for MCA diastolic coherence (*F*_1,43_ = 10.6; *p* = 0.002; $\eta_{G}^{2}$ = 0.20 [moderate]), MCA mean coherence (*F*_1,44_ = 7.27; *p* = 0.010; $\eta_{G}^{2}$ = 0.14 [moderate]), MCA systolic coherence (*F*_1,42_ = 5.56; *p* = 0.023; $\eta_{G}^{2}$ = 0.12 [small]), PCA diastolic coherence (*F*_1,43_ = 8.97; *p* = 0.005; $\eta_{G}^{2}$ = 0.17 [moderate]), and PCA mean coherence (*F*_1,44_ = 5.82; *p* = 0.020; $\eta_{G}^{2}$ = 0.12 [small]; Figures 4 and 5). Sex main effects were present at 0.10 Hz for MCA diastolic gain (*F*_1,43_ = 4.59; *p* = 0.038; $\eta_{G}^{2}$ = 0.10 [small]), MCA mean gain (*F*_1,44_ = 8.13; *p* = 0.007; $\eta_{G}^{2}$ = 0.16 [moderate]), MCA systolic gain (*F*_1,42_ = 17.6; *p* < 0.001; $\eta_{G}^{2}$ = 0.30 [large]), MCA systolic normalized gain (*F*_1,42_ = 10.1; *p* *= 0.002*; $\eta_{G}^{2}$ = 0.21 [moderate]), PCA diastolic gain (*F*_1,43_ = 4.59; *p* = 0.038; $\eta_{G}^{2}$ = 0.10 [small]), PCA mean gain (*F*_1,44_ = 5.26; *p* = 0.027; $\eta_{G}^{2}$ = 0.11 [small]), PCA systolic phase (*F*_1,43_ = 5.25; *p* = 0.027; $\eta_{G}^{2}$ = 0.11 [small]), PCA systolic gain (*F*_1,43_ = 18.2; *p* < 0.001; $\eta_{G}^{2}$ = 0.30 [large]), and PCA systolic normalized gain (*F*_1,43_ = 14.4; *p* < 0.001; $\eta_{G}^{2}$ = 0.25 [moderate]; Figures 4 and 5). Across all aspects of the cardiac cycle within both vessels and at 0.05 and 0.10 Hz, all other omnibus tests (type, sex, and type by sex) were not significant (all *p* > 0.067) and displayed negligible or small effect sizes ($\eta_{G}^{2}$ < 0.08; Figures 2, 3, 4, 5).

### 3.5 Dynamic cerebral autoregulation pairwise comparisons

Compared to their male counterparts, the pairwise comparisons revealed females had greater: 0.05 Hz MCA systolic gain (*p* = 0.003; Cohen's *d* = 0.97 [large]), 0.10 Hz MCA diastolic gain (*p* = 0.045; Cohen's *d* = 0.62 [moderate]), 0.10 Hz MCA mean gain (*p* = 0.007; Cohen's *d* = 0.84 [large]), 0.10 Hz MCA systolic gain (*p* < 0.001; Cohen's *d* = 1.26 [large]), 0.10 Hz MCA systolic normalized gain (*p* = 0.002; Cohen's *d* = 0.99 [large]), 0.10 Hz PCA diastolic gain (*p* = 0.038; Cohen's *d* = 0.62 [moderate]), 0.10 Hz PCA mean gain (*p* = 0.027; Cohen's *d* = 0.67 [moderate]), 0.10 Hz PCA systolic gain (*p* < 0.001; Cohen's *d* = 1.22 [large]), and 0.10 Hz PCA systolic normalized gain (*p* < 0.001; Cohen's *d* = 1.07 [large]); but lower values for: 0.05 Hz MCA systolic coherence (*p* = 0.045; Cohen's *d* = 0.61 [moderate]), 0.05 Hz PCA systolic coherence (*p* = 0.018; Cohen's *d* = 0.75 [moderate]), and 0.10 Hz PCA systolic phase (*p* = 0.027; Cohen's *d* = 0.66 [moderate]; Figures 2, 3, 4, 5). Compared to the bodyweight squats, the rSSM variables were greater for: 0.05 Hz MCA diastolic coherence (*p* = 0.014; Cohen's *d* = 0.76 [moderate]), 0.05 Hz MCA mean coherence (*p* = 0.049; Cohen's *d* = 0.61 [moderate]), 0.05 Hz PCA diastolic coherence (*p* = 0.07; Cohen's *d* = 0.84 [large]), 0.05 Hz PCA mean coherence (*p* = 0.045; Cohen's *d* = 0.62 [moderate]), 0.10 Hz MCA diastolic coherence (*p* = 0.002; Cohen's *d* = 0.96 [large]), 0.10 Hz MCA mean coherence (*p* = 0.010; Cohen's *d* = 0.78 [moderate]), 0.10 Hz MCA systolic coherence (*p* = 0.023; Cohen's *d* = 0.69 [moderate]), 0.10 Hz PCA diastolic coherence (*p* = 0.005; Cohen's *d* = 0.88 [large]), and 0.10 Hz PCA mean coherence (*p* = 0.020; Cohen's *d* = 0.78 [moderate]; Figures 2, 3, 4, 5).

## 4 DISCUSSION

This study sought to understand the autoregulatory capabilities of the cerebrovasculature during bodyweight and rSSM across the cardiac cycle, while also comparing males and females to understand the impact chromosomal sex has on the cerebral pressure‐flow relationship during resistance exercise. The current findings indicated rSSM induced greater oscillations in BP, as demonstrated by the augmented absolute MAP and diastolic BP values compared to bodyweight SSM at 0.05 and 0.10 Hz. These changes were only translated into the PSD at 0.10 Hz, where task and task by sex main effects were present for certain variables (Table 2). Nevertheless, the MCA and PCA PSD were not significant across the cardiac cycle at either frequency, indicating the cerebrovasculature was able to effectively buffer the oscillations in BP induced by both resisted and bodyweight SSM. When comparing between sexes, females displayed attenuated dCA metrics that were most notably associated with the systolic cardiac cycle component. Finally, when comparing between 0.05 and 0.10 Hz, more differences were noted between tasks and sexes within the latter. This increased buffering observed at 0.05 Hz is consistent with the broader literature, as with SSM performed at 0.05 Hz, the baroreflex has time to contribute to the cerebral pressure‐flow relationship (Zhang et al., 2009), thus mitigating against various confounding influences (e.g., type of squat, chromosomal sex, etc.).

### 4.1 Differences in SSM and rSSM

Squat‐stand maneuvers are a form of dynamic exercise capable of inducing a cyclical pressor response of ~30–50 mmHg fluctuations in MAP (Smirl et al., 1985). However, with resistance exercise (e.g., maximal squats/leg press), three to four‐fold increases in systemic BP have been reported (MacDougall et al., 1985; Palatini et al., 1989). Nevertheless, this type of exercise is only capable of being sustained for a few repetitions (~10–20 s maximum; Hargreaves & Spriet, 2020), which is substantially less than the duration required to obtain valid and reliable TFA estimates (~5‐min; Burma et al., 2021). Therefore, a trade‐off exists regarding the ability to examine the cerebral pressure‐flow relationship during low‐level resistance exercise, as an individual must be able to sustain this additional level of exertion/stress for a prolonged period (~5‐min). However, the novel methodological approach within the current investigation enabled the investigators to comprehensively assess the cerebrovascular regulatory capabilities *during* an augmented stressor.

Within the current investigation, it was found the rSSM elevated diastolic BP and MAP; however, systolic BP was comparable between tasks (Table 1). More so, the PSD demonstrated a task and/or task by sex main effect for the BP PSD at 0.10 Hz, but not at 0.05 Hz (Table 2). Opposingly, no differences were noted between tasks for both the MCA and PCA PSD between tasks (Table 2). The collection of these findings highlights three important observations. First, the rSSM induced a greater BP response which required the cerebrovasculature to dampen this increased stressor (Table 2). Second, despite this increased level of stress on the cerebrovasculature, the brain effectively buffered these BP elevations as evidenced by the lack of differences between tasks within MCA PSD, PCA PSD, and all TFA outcome metrics, aside from coherence parameters (which greatly exceeded the a priori coherence threshold of 0.46; Table 2 and Figures 2, 3, 4, 5). Third, the presence of an elevated BP PSD at 0.10 Hz but not at 0.05 Hz is indicative of greater BP buffering associated with slower frequency SSM, highlighting the inherent mechanisms of autoregulation (Table 2). For example, the brain acts as a high‐pass filter, effectively buffering slower frequency oscillations, whereas higher‐frequency oscillations are able to pass through to the cerebrovasculature relatively unimpeded (Zhang et al., 1998). However, with a given change in BP, it takes the baroreflex response ~4–7 s to modulate the BP fluctuation, which corresponds to a frequency of ~0.07–0.13 Hz (Zhang et al., 2009). As such, SSM performed at 0.10 Hz would only engage the baroreflex to modulate the induced oscillations in MAP in individuals with a greater fitness status (Besnier et al., 2017). Contrarily, given the SSM at 0.05 Hz is performed below the aforementioned frequency range, the baroreflex would be activated in the vast majority of individuals, regardless of fitness status. Therefore, the greater regulation capacity (both dCA and baroreflex modulated) at 0.05 Hz could be an explanation for the underlying mechanism as to why no task and/or task by sex difference in PSD were found at 0.05 Hz, whereas differences were noted at 0.10 Hz (Table 2).

### 4.2 Differences between sexes

Previous research examining TFA metrics between sexes have produced somewhat equivocal findings (Burma et al., 2020a; Favre & Serrador, 2019; Labrecque, Rahimaly, et al., 2019), For example, Burma et al. (2020a) demonstrated no differences between chromosomal sexes in all TFA metrics across all aspects of the cardiac cycle. Conversely, Labrecque, Rahimaly, et al. (2019) found highly trained females exhibited less autoregulatory abilities compared to their male counterparts. However, Favre and Serrador (2019) unveiled females had greater regulation present at three different time points across the menstrual cycle. The divergent findings can likely be attributable to the differential methodology utilized. For example, Burma et al. (2020a) examined females within days 3–7 of the menstrual cycle with SSM at both 0.05 and 0.10 Hz, albeit there was no control for cardiorespiratory fitness. Likewise, Labrecque, Rahimaly, et al. (Labrecque, Rahimaly, et al., 2019) conducted SSM within days 1–10 of the menstrual cycle for females at 0.05 and 0.10 Hz, while additionally controlling for cardiorespiratory fitness by having all participants perform a maximal oxygen uptake test. Finally, Favre and Serrador (2019) had participants perform SSM at three time points across the menstrual cycle (early follicular phase [day 3 ± 1], late follicular phase [day 13 ± 2], and mid‐luteal phase [23 ± 3]); however, these were only conducted at 0.05 Hz and similar to Burma et al. (2020a) there was no control associated with cardiorespiratory fitness. Furthermore, only the study by Burma et al. (2020a) examined the cerebral pressure‐flow relationship within the diastolic and systolic aspects of the cardiac cycle.

The current investigation is primarily in alignment with the findings by Labrecque, Rahimaly, et al. (2019) as females demonstrated numerous augmented gain values compared to males and a lower phase in the PCA at 0.10 Hz (Figures 2, 3, 4, 5). However, the vast majority of differences between chromosomal sexes were noted within the systolic aspect of the cardiac cycle compared to the diastolic or mean components (Favre & Serrador, 2019; Labrecque, Rahimaly, et al., 2019). This may explain why the findings by Labrecque, Rahimaly, et al. (2019) found subtle differences between males and females with the SSM, as they only examined the mean component of the cardiac cycle. Additionally, it cannot be ruled out the differences were in part due to changes in hormone concentrations across the menstrual cycle, as the day of testing was not controlled (Shechter & Boivin, 2010). While Favre and Serrador (2019) demonstrated no impact of the menstrual cycle on TFA estimates, these authors only examined mean metrics during SSM at 0.05 Hz. Conversely, the present investigation, highlights the majority of differences were at 0.10 Hz in the systolic phase (Figures 4 and 5). Therefore, future research is needed to comprehensively examine the cerebral pressure‐flow relationship, with regard to cardiorespiratory fitness and the menstrual cycle.

The findings highlight imperative clinical findings, that may lie at the helm of cerebrovascular accidents and alterations with a concussion. For example, a plethora of findings has demonstrated that males have a greater incidence of stroke compared to females (Appelros et al., 2009; Reeves et al., 2008; Vyas et al., 2021). However, due to the longer life expectancy for the latter, females have a greater total number of strokes, that are of greater severity (Reeves et al., 2008). Of note, a prospective cohort study consisting of 9.2 million individuals, found the hazard ratio for females aged 18–30 was greater for both ischemic stroke (1.47; 95% CI: 1.19–1.83) and transient ischemic attacks (2.02; 95% CI: 1.51–2.72; Vyas et al., 2021). However, at this age, there are no differences between males and females with regard to subarachnoid hemorrhage (0.98; 95% CI: 0.69–1.40; Vyas et al., 2021). Conversely, for ischemic and transient ischemic attacks, the risk is greater for females aged 40–79; however, subarachnoid hemorrhages are greater for females from 30 onwards (Vyas et al., 2021). Therefore, as the present investigation included females within the 18–30 age range, the findings may demonstrate a mechanistic understanding of why the risk of ischemic attacks are greater for females in this age range. For example, dCA describes the ability of the brain to maintain adequate perfusion despite transient changes in BP (Brassard et al., 2021; Paulson et al., 1990; Zhang et al., 1998). If the brain has attenuated autoregulation, it may not receive adequate perfusion, and thus could be put at an elevated risk of transient ischemic attacks (Aries et al., 2010; Lidington et al., 2021). Nevertheless, the primary sex difference was impaired systolic gain, which refers to the extent the vasculature expanded with each systolic peak. Hence, the findings are more likely a physiological marker for the augmented risk of the elevation of subarachnoid hemorrhage within females (Lidington et al., 2021), a brain injury defined as bleeding between the brain and subarachnoid space, most often caused by a weakening of the arterial wall. This injury does not have a rapid onset but rather occurs slowly over time with a gradual weakening of the vasculature, commonly resulting from ruptured aneurysms (Marcolini & Hine, 2019). Therefore, while the sample utilized had females aged 25 ± 4 years, the long‐term consequences of impaired systolic gain could translate to the clinically greater hazard for subarachnoid hemorrhage for females >30 years.

### 4.3 Implications for future research

While the current investigations revealed novel findings, it also unveiled numerous implications for future research investigations. First, the vast majority of previous investigations using TCD have only examined one phase of the cardiac cycle independently (primarily mean), rather than examining all three aspects (i.e., diastole, mean, and systole) comprehensively. By only examining one phase, research may miss out on subtle differences that could delineate chromosomal sexes, clinical groups, and so forth (Wright, Smirl, Bryk, Fraser, et al., 2018). Second, investigations utilizing SSM to quantify the cerebral pressure‐flow relationship should use both 0.05 and 0.10 Hz as they reveal differences in autoregulatory capabilities (Smirl et al., 1985). For example, with respect to task, greater differences were found at 0.10 Hz due to these SSM occurring too quickly for baroreflex mediated changes to occur. Third, continuing work is required to delineate how TFA metrics are confounded individually and/or jointly by cardiorespiratory fitness, chromosomal sex, phase of the menstrual cycle, and hormone concentrations (Labrecque, Rahimaly, et al., 2019; Labrecque et al., 2017; Labrecque Smirl et al., 2019). Fourth, future research is warranted to understand how these findings of impaired autoregulation in females relate to the physiological underpinnings of various clinical populations (e.g., cerebrovascular accidents, concussion, etc.). For example, within the realm of concussion, aerobic treadmill and cycle tests (e.g., Buffalo Concussion Treadmill/Bike Test; Graham et al., 2021; Haider et al., 2019) have been developed to assess physiological recovery following concussion and the readiness to perform resistance‐based training exercises as recommended in stage four of the current return‐to‐play concussion guidelines (McCrory et al., 2017). The present methodology and findings may demonstrate a potential and feasible way to assess an individual's readiness to return to resistance training following concussion. However, further research is warranted to confirm this proposition.

### 4.4 Limitations

While an adequate number of subjects were recruited based upon the a priori sample size calculation, the attrition due to technological difficulties with the rSSM reduced the total sample for the interaction comparisons. This means the present study could have been slightly underpowered to determine the type of squat by sex interaction differences (Type II error). Nevertheless, interaction main effects were found within the blood pressure PSD minimizing the concerns surrounding the power of the present study. Additionally, all interaction main effects for all TFA metrics were *p* > 0.114 with small/negligible effect sizes (all $\eta_{G}^{2}$ ≤ 0.06). Therefore, given the differences found for the within, between, and interaction comparisons, the findings are likely highly representative of physiological differences between the type of squat performed and between chromosomal sexes.

The largest limitation surrounding the use of TCD is due to the fact it is unable to measure vessel diameter and thus relies on the assumption that this remains stable (Ainslie & Hoiland, 1985). Previous research using functional magnetic resonance imaging has demonstrated CBV remains stable as long as end‐tidal values remain with ~8 mmHg of eucapnic values (Coverdale et al., 1985; Verbree et al., 1985). During all SSM, P_ET_CO_2_ values were closely monitored where breathing was coached to ensure these did not impact the outcome results. Table 2 highlights P_ET_CO_2_ values were within ~1 mmHg across all tasks between sexes, which displayed minimal CoV (<5%). Therefore, differences in the outcome measures due to carbon dioxide influences had a nominal impact (Yoshida et al., 2018). When feasible, future studies should seek to use methods capable of clamping end‐tidal values to completely mitigate the likelihood respiratory and carbon dioxide parameters would impact TFA estimates. Furthermore, as previously described, it is unaware how menstrual cycle and cardiorespiratory fitness status impacted the outcome metrics of interest. However, this would only impact the sex and task by sex comparisons, as each individual acted as their own control where they performed all four SSM. Thus, all confounding factors would be accounted for within the task comparisons, as each individual acted as their own control (Mills et al., 2009).

## 5 CONCLUSION

The aim of the current study was the understand the capabilities of the cerebral pressure‐flow relationship during elevated stress (i.e., bodyweight vs. resistance weight SSM), while also comparing potential differences between chromosomal sexes. The findings demonstrated rSSM elevated absolute diastolic and mean BP at both 0.05 and 0.10 Hz; however, task effects were only found at 0.10 Hz regarding PSD metrics. Despite the augmented perfusion pressure within the brain, no differences were noted within phase, gain, and normalized gain metrics between tasks, indicating the brain was efficient at dampening this augmented stressor. Furthermore, greater differences were seen between sexes/genders in the systolic aspect of the cardiac cycle at 0.10 Hz during both bodyweight and rSSM. Conclusively, these may have important clinical applications that may explain the physiological underpinnings of various conditions/diseases. Nonetheless, future research is warranted to delineate the associations between these findings and potential clinical presentations.

## CONFLICT OF INTEREST

The authors declare they have no conflicts of interest to report.

## ETHICAL APPROVAL

The Conjoint Health Research Ethics Board at the University of Calgary approved this study (REB20‐1662 and REB20‐2112). Data were collected in July of 2021, where all subjects gave written informed consent prior to data collection and protocols proceeded according to institutional guidelines.

## AUTHOR CONTRIBUTION

**Kailey T. Newel:**Conceptualization, Methodology Investigation, Writing – Original draft, Writing – Review & Editing; **Joel S. Burma:** Conceptualization, Methodology, Formal analysis, Investigation, Writing – Original draft, Writing – Review & Editing, Visualization; **Joseph Carere:** Investigation, Writing – original draft, Writing – Review & Editing; **Courtney M. Kennedy:** Conceptualization, Methodology, Writing – original draft, Writing – Review & Editing; **Jonathan D. Smirl:** Conceptualization, Resources, Writing – Review & Editing, Supervision, Funding acquisition.

## References

[phy215278-bib-0001] Ainslie, P. N. , & Hoiland, R. L. (1985). Transcranial Doppler ultrasound: Valid, invalid, or both? Journal of Applied Physiology, 117(10), 1081–1083. 10.1152/japplphysiol.00854.2014 25257879

[phy215278-bib-0002] Ainslie, P. N. , Murrell, C. , Peebles, K. , Swart, M. , Skinner, M. A. , Williams, M. J. , & Taylor, R. D. (2007). Early morning impairment in cerebral autoregulation and cerebrovascular CO_2_ reactivity in healthy humans: Relation to endothelial function. Experimental Physiology, 92, 769–777. 17384117 10.1113/expphysiol.2006.036814

[phy215278-bib-0003] Amrhein, V. , Greenland, S. , & McShane, B. (2019). Scientists rise up against statistical significance. Nature, 567, 305–307. 10.1038/d41586-019-00857-9 30894741

[phy215278-bib-0004] Appelros, P. , Stegmayr, B. , & Terént, A. (2009). Sex differences in stroke epidemiology. Stroke, 40, 1082–1090. 10.1161/STROKEAHA.108.540781 19211488

[phy215278-bib-0005] Aries, M. J. , Elting, J. W. , De Keyser, J. , Kremer, B. P. , & Vroomen, P. C. (2010). Cerebral autoregulation in stroke: A review of transcranial Doppler studies. Stroke, 41, 2697–2704. 20930158 10.1161/STROKEAHA.110.594168

[phy215278-bib-0006] Atkinson, G. , & Nevill, A. M. (1998). Statistical methods for assessing measurement error (reliability) in variables relevant to sports medicine. Sports Medicine, 26, 217–238. 10.2165/00007256-199826040-00002 9820922

[phy215278-bib-0007] Bakeman, R. (2005). Recommended effect size statistics for repeated measures designs. Behavior Research Methods, 37, 379–384. 10.3758/BF03192707 16405133

[phy215278-bib-0008] Besnier, F. , Labrunée, M. , Pathak, A. , Pavy‐Le Traon, A. , Galès, C. , Sénard, J. M. , & Guiraud, T. (2017). Exercise training‐induced modification in autonomic nervous system: An update for cardiac patients. Annals of Physical and Rehabilitation Medicine, 60, 27–35. 27542313 10.1016/j.rehab.2016.07.002

[phy215278-bib-0009] Blanca, M. J. , Alarcón, R. , Arnau, J. , Bono, R. , & Bendayan, R. (2017). Non‐normal data: Is ANOVA still a valid option? Psicothema, 29, 552–557. 29048317 10.7334/psicothema2016.383

[phy215278-bib-0010] Brassard, P. , Labrecque, L. , Smirl, J. D. , Tymko, M. M. , Caldwell, H. G. , Hoiland, R. L. , Lucas, S. J. E. , Denault, A. Y. , Couture, E. J. , & Ainslie, P. N. (2021). Losing the dogmatic view of cerebral autoregulation. Physiological Reports, 9, e14982. 10.14814/phy2.14982 34323023 PMC8319534

[phy215278-bib-0011] Burma, J. S. , Copeland, P. V. , Macaulay, A. , Khatra, O. , & Smirl, J. D. (2020a). Comparison of diurnal variation, anatomical location, and biological sex within spontaneous and driven dynamic cerebral autoregulation measures. Physiol Rep, 8, e14458. 10.14814/phy2.14458 32537905 PMC7293969

[phy215278-bib-0012] Burma, J. S. , Copeland, P. V. , Macaulay, A. , Khatra, O. , & Smirl, J. D. (2020b). Effects of high‐intensity intervals and moderate‐intensity exercise on baroreceptor sensitivity and heart rate variability during recovery. Applied Physiology, Nutrition, and Metabolism, 45, 1156–1164. 10.1139/apnm-2019-0810 32343909

[phy215278-bib-0013] Burma, J. S. , Copeland, P. , Macaulay, A. , Khatra, O. , Wright, A. D. , & Smirl, J. D. (2020). Dynamic cerebral autoregulation across the cardiac cycle during 8 hr of recovery from acute exercise. Physiol Rep, 8, e14367. 10.14814/phy2.14367 32163235 PMC7066871

[phy215278-bib-0014] Burma, J. S. , Miutz, L. N. , Newel, K. T. , Labrecque, L. , Drapeau, A. , Brassard, P. , Copeland, P. , Macaulay, A. , & Smirl, J. D. (2021). What recording duration is required to provide physiologically valid and reliable dynamic cerebral autoregulation transfer functional analysis estimates? Physiological Measurement, 42. 10.1088/1361-6579/abf1af 33761474

[phy215278-bib-0017] Claassen, J. A. , Levine, B. D. , & Zhang, R. (2009). Dynamic cerebral autoregulation during repeated squat‐stand maneuvers. Journal of Applied Physiology, 106(1), 153–160. 10.1152/japplphysiol.90822.2008 18974368 PMC2636935

[phy215278-bib-0018] Claassen, J. A. , Meel‐van den Abeelen, A. S. , Simpson, D. M. , & Panerai, R. B. (2016). Transfer function analysis of dynamic cerebral autoregulation: A white paper from the International Cerebral Autoregulation Research Network. Journal of Cerebral Blood Flow and Metabolism, 36, 665–680. 26782760 10.1177/0271678X15626425 PMC4821028

[phy215278-bib-0019] Claassen, J. , Thijssen, D. H. J. , Panerai, R. B. , & Faraci, F. M. (2021). Regulation of cerebral blood flow in humans: Physiology and clinical implications of autoregulation. Physiological Reviews, 101, 1487–1559. 33769101 10.1152/physrev.00022.2020 PMC8576366

[phy215278-bib-0020] Coverdale, N. S. , Gati, J. S. , Opalevych, O. , Perrotta, A. , & Shoemaker, J. K. (1985). Cerebral blood flow velocity underestimates cerebral blood flow during modest hypercapnia and hypocapnia. Journal of Applied Physiology, 117(10), 1090–1096. 10.1152/japplphysiol.00285.2014 25012027

[phy215278-bib-0021] Favre, M. E. , & Serrador, J. M. (2019). Sex differences in cerebral autoregulation are unaffected by menstrual cycle phase in young, healthy women. American Journal of Physiology. Heart and Circulatory Physiology, 316, H920–h933. 10.1152/ajpheart.00474.2018 30707610

[phy215278-bib-0022] Graham, R. F. , van Rassel, C. R. , Burma, J. S. , Rutschmann, T. D. , Miutz, L. N. , Sutter, B. , & Schneider, K. J. (2021). Concurrent validity of a stationary cycling test and buffalo concussion treadmill test in adults with concussion. Journal of Athletic Training. 10.4085/1062-6050-0003.21 PMC8675311 34911073

[phy215278-bib-0023] Haider, M. N. , Leddy, J. J. , Wilber, C. G. , Viera, K. B. , Bezherano, I. , Wilkins, K. J. , Miecznikowski, J. C. , & Willer, B. S. (2019). The predictive capacity of the buffalo concussion treadmill test after sport‐related concussion in adolescents. Frontiers in Neurology, 10. 10.3389/fneur.2019.00395 PMC6492460 31105634

[phy215278-bib-0024] Hamner, J. W. , & Tan, C. O. (2014). Relative contributions of sympathetic, cholinergic, and myogenic mechanisms to cerebral autoregulation. Stroke, 45, 1771–1777. 10.1161/STROKEAHA.114.005293 24723314 PMC4102642

[phy215278-bib-0025] Hamner, J. W. , Tan, C. O. , Lee, K. , Cohen, M. A. , & Taylor, J. A. (2010). Sympathetic control of the cerebral vasculature in humans. Stroke, 41, 102–109. 10.1161/STROKEAHA.109.557132 20007920 PMC2814242

[phy215278-bib-0026] Hargreaves, M. , & Spriet, L. L. (2020). Skeletal muscle energy metabolism during exercise. Nature Metabolism, 2, 817–828. 10.1038/s42255-020-0251-4 32747792

[phy215278-bib-0027] Jager, K. J. , Zoccali, C. , MacLeod, A. , & Dekker, F. W. (2008). Confounding: What it is and how to deal with it. Kidney International, 73, 256–260. 17978811 10.1038/sj.ki.5002650

[phy215278-bib-0028] Labrecque, L. , Rahimaly, K. , Imhoff, S. , Paquette, M. , Le Blanc, O. , Malenfant, S. , Drapeau, A. , Smirl, J. D. , Bailey, D. M. , & Brassard, P. (2019). Dynamic cerebral autoregulation is attenuated in young fit women. Physiological Reports, 7, e13984. 10.14814/phy2.13984 30652420 PMC6335382

[phy215278-bib-0029] Labrecque, L. , Rahimaly, K. , Imhoff, S. , Paquette, M. , Le Blanc, O. , Malenfant, S. , Lucas, S. J. E. , Bailey, D. M. , Smirl, J. D. , & Brassard, P. (2017). Diminished dynamic cerebral autoregulatory capacity with forced oscillations in mean arterial pressure with elevated cardiorespiratory fitness. Physiological Reports, 5. 10.14814/phy2.13486 PMC5688778 29122957

[phy215278-bib-0030] Labrecque, L. , Smirl, J. D. , & Brassard, P. (2019). Letter to the Editor: On the need of considering cardiorespiratory fitness when examining the influence of sex on dynamic cerebral autoregulation. American Journal of Physiology. Heart and Circulatory Physiology, 316, H1229. 31070459 10.1152/ajpheart.00152.2019

[phy215278-bib-0031] Lakens, D. (2013). Calculating and reporting effect sizes to facilitate cumulative science: A practical primer for *t*‐tests and ANOVAs. Frontiers in Psychology, 4, 863. 24324449 10.3389/fpsyg.2013.00863 PMC3840331

[phy215278-bib-0032] Lefrandt, J. D. , Mulder, M. C. , Bosma, E. , Smit, A. J. , & Hoogenberg, K. (2000). Inverse relationship between blood glucose and autonomic function in healthy subjects. Diabetes Care, 23, 1862–1864. 10.2337/diacare.23.12.1862 11128375

[phy215278-bib-0033] Lidington, D. , Wan, H. , & Bolz, S.‐S. (2021). Cerebral autoregulation in subarachnoid hemorrhage. Frontiers in Neurology, 12. 10.3389/fneur.2021.688362 PMC8342764 34367053

[phy215278-bib-0034] MacDougall, J. D. , Tuxen, D. , Sale, D. G. , Moroz, J. R. , & Sutton, J. R. (1985). Arterial blood pressure response to heavy resistance exercise. Journal of Applied Physiology, 58, 785–790. 10.1152/jappl.1985.58.3.785 3980383

[phy215278-bib-0035] Maclure, M. (1991). The case‐crossover design: A method for studying transient effects on the risk of acute events. American Journal of Epidemiology, 133, 144–153. 1985444 10.1093/oxfordjournals.aje.a115853

[phy215278-bib-0036] Marcolini, E. , & Hine, J. (2019). Approach to the diagnosis and management of subarachnoid hemorrhage. Western Journal of Emergency Medicine, 20, 203–211. 10.5811/westjem.2019.1.37352 30881537 PMC6404699

[phy215278-bib-0037] McCrory, P. , Meeuwisse, W. , Dvořák, J. , Aubry, M. , Bailes, J. , Broglio, S. , Cantu, R. C. , Cassidy, D. , Echemendia, R. J. , Castellani, R. J. , Davis, G. A. , Ellenbogen, R. , Emery, C. , Engebretsen, L. , Feddermann‐Demont, N. , Giza, C. C. , Guskiewicz, K. M. , Herring, S. , Iverson, G. L. , … Vos, P. E. (2017). Consensus statement on concussion in sport‐the 5(th) international conference on concussion in sport held in Berlin, October 2016. British Journal of Sports Medicine, 51, 838–847. 28446457 10.1136/bjsports-2017-097699

[phy215278-bib-0038] Mills, E. J. , Chan, A. W. , Wu, P. , Vail, A. , Guyatt, G. H. , & Altman, D. G. (2009). Design, analysis, and presentation of crossover trials. Trials, 10, 27. 10.1186/1745-6215-10-27 19405975 PMC2683810

[phy215278-bib-0039] Omboni, S. , Parati, G. , Frattola, A. , Mutti, E. , Di Rienzo, M. , Castiglioni, P. , & Mancia, G. (1993). Spectral and sequence analysis of finger blood pressure variability. Comparison with analysis of intra‐arterial recordings. Hypertension, 22, 26–33. 10.1161/01.HYP.22.1.26 8319990

[phy215278-bib-0040] Palatini, P. , Mos, L. , Munari, L. , Valle, F. , Del Torre, M. , Rossi, A. , Varotto, L. , Macor, F. , Martina, S. , Pessina, A. C. , & Palù, C. D. (1989). Blood pressure changes during heavy‐resistance exercise. Journal of Hypertension. Supplement, 7, S72–S73. 10.1097/00004872-198900076-00032 2632751

[phy215278-bib-0041] Panagiotakos, D. B. (2008). Value of p‐value in biomedical research. Open Cardiovascular Medicine Journal, 2, 97–99. 19430522 10.2174/1874192400802010097 PMC2627527

[phy215278-bib-0042] Paulson, O. B. , Strandgaard, S. , & Edvinsson, L. (1990). Cerebral autoregulation. Cerebrovascular and Brain Metabolism Reviews, 2, 161–192. 2201348

[phy215278-bib-0043] Reeves, M. J. , Bushnell, C. D. , Howard, G. , Gargano, J. W. , Duncan, P. W. , Lynch, G. , Khatiwoda, A. , & Lisabeth, L. (2008). Sex differences in stroke: Epidemiology, clinical presentation, medical care, and outcomes. The Lancet Neurology, 7, 915–926. 18722812 10.1016/S1474-4422(08)70193-5 PMC2665267

[phy215278-bib-0044] Sammons, E. L. , Samani, N. J. , Smith, S. M. , Rathbone, W. E. , Bentley, S. , Potter, J. F. , & Panerai, R. B. (2007). Influence of noninvasive peripheral arterial blood pressure measurements on assessment of dynamic cerebral autoregulation. Journal of Applied Physiology, 103(1), 369–375. 10.1152/japplphysiol.00271.2007 17463300

[phy215278-bib-0045] Schielzeth, H. , Dingemanse, N. J. , Nakagawa, S. , Westneat, D. F. , Allegue, H. , Teplitsky, C. , Réale, D. , Dochtermann, N. A. , Garamszegi, L. Z. , & Araya‐Ajoy, Y. G. (2020). Robustness of linear mixed‐effects models to violations of distributional assumptions. Methods in Ecology and Evolution, 11, 1141–1152. 10.1111/2041-210X.13434

[phy215278-bib-0046] Shechter, A. , & Boivin, D. B. (2010). Sleep, hormones, and circadian rhythms throughout the menstrual cycle in healthy women and women with premenstrual dysphoric disorder. International Journal of Endocrinology, 2010, 1–17. 10.1155/2010/259345 PMC2817387 20145718

[phy215278-bib-0047] Simpson, D. , & Claassen, J. (2018). CrossTalk opposing view: Dynamic cerebral autoregulation should be quantified using induced (rather than spontaneous) blood pressure fluctuations. The Journal of Physiology, 596, 7–9. 29207208 10.1113/JP273900 PMC5746528

[phy215278-bib-0048] Smirl, J. D. , Hoffman, K. , Tzeng, Y. C. , Hansen, A. , & Ainslie, P. N. (1985). Methodological comparison of active‐ and passive‐driven oscillations in blood pressure; implications for the assessment of cerebral pressure‐flow relationships. Journal of Applied Physiology, 119(5), 487–501. 10.1152/japplphysiol.00264.2015 PMC4556839 26183476

[phy215278-bib-0049] Smirl, J. D. , Wright, A. D. , Ainslie, P. N. , Tzeng, Y. C. , & van Donkelaar, P. (2018). Differential systolic and diastolic regulation of the cerebral pressure‐flow relationship during squat‐stand manoeuvres. Acta Neurochirurgica. Supplementum, 126, 263–268. 10.1007/978-3-319-65798-1_52 29492572

[phy215278-bib-0050] Tan, C. O. , Hamner, J. W. , & Taylor, J. A. (2013). The role of myogenic mechanisms in human cerebrovascular regulation. Journal of Physiology, 591, 5095–5105. 10.1113/jphysiol.2013.259747 23959681 PMC3810812

[phy215278-bib-0051] Thiese, M. S. , Ronna, B. , & Ott, U. (2016). P value interpretations and considerations. Journal of Thoracic Disease, 8, E928–E931. 10.21037/jtd.2016.08.16 27747028 PMC5059270

[phy215278-bib-0052] van Baak, M. A. (2008). Meal‐induced activation of the sympathetic nervous system and its cardiovascular and thermogenic effects in man. Physiology & Behavior, 94, 178–186. 10.1016/j.physbeh.2007.12.020 18281067

[phy215278-bib-0053] Verbree, J. , Bronzwaer, A. S. , Ghariq, E. , Versluis, M. J. , Daemen, M. J. , van Buchem, M. A. , Dahan, A. , van Lieshout, J. J. , & van Osch, M. J. (1985). Assessment of middle cerebral artery diameter during hypocapnia and hypercapnia in humans using ultra‐high‐field MRI. Journal of Applied Physiology, 117(10), 1084–1089. 10.1152/japplphysiol.00651.2014 25190741

[phy215278-bib-0054] Vyas, M. V. , Silver, F. L. , Austin, P. C. , Yu, A. Y. X. , Pequeno, P. , Fang, J. , Laupacis, A. , & Kapral, M. K. (2021). Stroke Incidence by sex across the lifespan. Stroke, 52, 447–451. 10.1161/STROKEAHA.120.032898 33493057

[phy215278-bib-0055] Williams, L. R. , & Leggett, R. W. (1989). Reference values for resting blood flow to organs of man. Clinical Physics and Physiological Measurement, 10, 187–217. 10.1088/0143-0815/10/3/001 2697487

[phy215278-bib-0056] Willie, C. K. , Colino, F. L. , Bailey, D. M. , Tzeng, Y. C. , Binsted, G. , Jones, L. W. , Haykowsky, M. J. , Bellapart, J. , Ogoh, S. , Smith, K. J. , Smirl, J. D. , Day, T. A. , Lucas, S. J. , Eller, L. K. , & Ainslie, P. N. (2011). Utility of transcranial Doppler ultrasound for the integrative assessment of cerebrovascular function. Journal of Neuroscience Methods, 196, 221–237. 10.1016/j.jneumeth.2011.01.011 21276818

[phy215278-bib-0057] Willie, C. K. , Tzeng, Y. C. , Fisher, J. A. , & Ainslie, P. N. (2014). Integrative regulation of human brain blood flow. The Journal of Physiology, 592, 841–859. 10.1113/jphysiol.2013.268953 24396059 PMC3948549

[phy215278-bib-0058] Wilson, M. H. (2016). Monro‐Kellie 2.0: The dynamic vascular and venous pathophysiological components of intracranial pressure. Journal of Cerebral Blood Flow and Metabolism, 36, 1338–1350. 27174995 10.1177/0271678X16648711 PMC4971608

[phy215278-bib-0059] Wright, A. D. , Smirl, J. D. , Bryk, K. , Fraser, S. , Jakovac, M. , & van Donkelaar, P. (2018). Sport‐related concussion alters indices of dynamic cerebral autoregulation. Frontiers in Neurology, 9, 196. 10.3389/fneur.2018.00196 29636724 PMC5880892

[phy215278-bib-0060] Wright, A. D. , Smirl, J. D. , Bryk, K. , & van Donkelaar, P. (2018). Systolic and diastolic regulation of the cerebral pressure‐flow relationship differentially affected by acute sport‐related concussion. Acta Neurochirurgica. Supplementum, 126, 303–308. 10.1007/978-3-319-65798-1_59 29492579

[phy215278-bib-0061] Yoshida, H. , Hamner, J. W. , Ishibashi, K. , & Tan, C. O. (2018). Relative contributions of systemic hemodynamic variables to cerebral autoregulation during orthostatic stress. Journal of Applied Physiology, 124, 321–329. 10.1152/japplphysiol.00700.2017 29025902

[phy215278-bib-0062] Zhang, R. , Claassen, J. A. , Shibata, S. , Kilic, S. , Martin‐Cook, K. , Diaz‐Arrastia, R. , & Levine, B. D. (2009). Arterial‐cardiac baroreflex function: Insights from repeated squat‐stand maneuvers. American Journal of Physiology: Regulatory, Integrative and Comparative Physiology, 297, R116–R123. 19420293 10.1152/ajpregu.90977.2008 PMC2711702

[phy215278-bib-0063] Zhang, R. , Zuckerman, J. H. , Giller, C. A. , & Levine, B. D. (1998). Transfer function analysis of dynamic cerebral autoregulation in humans. American Journal of Physiology: Heart and Circulatory Physiology, 274, H233–H241. 10.1152/ajpheart.1998.274.1.H233 9458872

